# Evaluation of the impact of refrigeration on next generation sequencing-based assessment of the canine and feline fecal microbiota

**DOI:** 10.1186/s12917-014-0230-7

**Published:** 2014-09-30

**Authors:** J Scott Weese, Mohammad Jalali

**Affiliations:** Department of Pathobiology and Centre for Public Health and Zoonoses, Ontario Veterinary College, University of Guelph, Guelph, ON N1G2W1 Canada

**Keywords:** Microbiota, Microflora, Intestinal, Gastroenterology

## Abstract

**Background:**

Evaluation of factors that might impact microbiota assessment is important to avoid spurious results, particularly in field and multicenter studies where sample collection may occur distant from the laboratory. This study evaluated the impact of refrigeration on next generation sequence-based assessment of the canine and feline fecal microbiota. Fecal samples were collected from seven dogs and ten cats, and analysed at baseline and after 3, 7, 10 and 14 days of storage at 4°C.

**Results:**

There were no differences in community membership or population structure between timepoints for either dogs or cats, nor were there any differences in richness, diversity and evenness. There were few differences in relative abundance of phyla or predominant genera, with the only differences being significant increases in Actinobacteria between Days 0-14 (*P =* 0.0184) and 1-14 (*P* = 0.0182) for canine samples, and a decrease in Erysipelotrichaceae *incertae sedis* between Day 0 and Day 7 (median 4.9 vs 2.2%, *P =* 0.046) in feline samples.

Linear discriminant analysis effect size and indicator analysis identified a small number of genera that were over-represented in, or defining characteristics of, Day 14 samples. These were predominantly Proteobacteria and Actinobacteria, with *Psychrobacter* and *Arthrobacter* enriched in both canine and feline Day 14 samples.

**Conclusions:**

Storage for at least 14 days at 4°C has limited impact on culture-independent assessment of the canine and feline fecal microbiota, although changes in some individual groups may occur.

## Background

Advances in next generation sequencing and bioinformatics have revolutionized the study of complex microbial populations. Recent studies in dogs and cats have characterized the fecal, intestinal, oral and skin microbiotas [1-5], and provided important insights into both the composition of the microbiota and its relationship with various diseases. However, microbiota assessment is not infallible and there are many steps in the process that could potentially impact results. One is the time from sample collection to processing, an important factor for field studies where samples may be collected remotely from the laboratory. An understanding of the impact of storage conditions and time is important for study design and interpretation.

There has been limited study of the impact of storage on the fecal microbiota. A study of human fecal samples reported limited impact of storage at room temperature for 24 h or at -80°C for six months [6]. Similarly little impact was identified in human vaginal samples stored at -20°C and -80°C [7]. A study of human and soil samples detected no influence of storage, including refrigeration, on microbial population structure or diversity, but some shifts in relative abundances of different taxa [8], results that were similar to a later study of soil bacteria [9].

Since field and multicenter studies often involve delays from sample collection to processing, it is critical to understand the potential impacts of storage. Refrigeration (4°C) is the most convenient temperature for temporary storage and shipping, and it is important to understand if short-term refrigeration will significantly impact downstream analysis. Species- and sample (i.e. body site)-specific study is required because of the differences in the microbiota between species and body sites, and the potential that there could be different impacts of storage on populations within these samples. The objective of this study was to evaluate the impact of refrigeration on assessment of the canine and feline fecal microbiota.

## Methods

Fecal samples were collected from seven dogs and ten cats from a local animal shelter immediately after defecation. Dogs were clinically normal with no history of antimicrobial exposure or gastrointestinal disease, although medical histories were limited based on the nature of the population. Samples were stored in plastic fecal containers at 4°C for up to 2 hours prior to arrival at the laboratory. Immediately after arrival, samples were manually homogenized and separated into five aliquots. One aliquot was processed immediately while the other four were stored in a refrigerator at 4°C. One of each aliquots was then tested after 3, 7, 10 and 14 days of refrigeration.

DNA was extracted using a commercial kit^a^, and DNA quantity and quality were assessed by spectrophotometry^b^. The V4 region of the 16S rRNA gene was then amplified using the primers S-D-Bact-0564-a-S-15 (5'-AYTGGGYDTAAAGNG-3') and S-D-Bact-0785-b-A-18 (5'-TACNVGGGTATCTAATCC-3') [10]. The amplicon library was purified with Agencourt AMPure XP beads^c^ with slight modification to the manufacturer’s protocol. Briefly, 72 μL of AMPure beads was added to 20 μL of library and incubated for 10 min at room temperature. Samples were washed twice with 80% ethanol, and eluted with 20 μL of PCR-grade H_2_O. Purified samples were quantified by spectrophotometry, evaluated by electrophoresis on a 1% agarose gel, and diluted to 5 ng/μL. Sequencing was performed using an Illumina MiSeq with 2X250 chemistry.^d^

Mothur v33.3 was used for analysis [11]. After paired end reads were assembled, sequences were aligned with the Silva 16S rRNA reference database [12] and any sequences not consistent with the target amplicon size (240 bp), containing any ambiguous base calls or long runs (>8 bp) of holopolymers, or that did not align with the correct 16S rRNA gene region were removed. Chimeras were detected using uchime [13] and removed. Taxonomy was assigned using the RDP taxonomy database [14]. Sequences were then binned into operational taxon units (OTUs) at a 3% dissimilarity level.

Subsampling was performed to normalize sequence number for analyses [15]. This involved random selection of a number of sequences from each sample that corresponded to the smallest number of sequences from an individual sample. Population diversity (inverse Simpson’s index), evenness (Shannon’s evenness index) and richness (Chao1) were calculated and compared between groups using Wilcoxon and Steel-Dwass tests. Linear discriminatory analysis effective size (LefSe) [16] analysis and indicator analysis [17] were performed.

Community membership was described using the classical Jaccard index, while population structure (evaluation of membership and relative abundance of members) was evaluated using the Yue & Clayton index of dissimilarity and Bray-Curtis index. Unifrac was used to compare these indices between groups [18]. Principal coordinate analysis (PCoA) and random forest analysis were also performed. The relative abundance of all phyla were compared between groups, along with the relative abundance of all genera accounting for ≥ 1% of sequences in the Day 0 or Day 14 samples using the Steel-Dwass test. A *P* <0.05 was considered significant for all comparisons.

## Results

A total of 3,722,656 sequences passed all quality control filters, with a median sequence count of 41970 per sample. Subsampling at 5177 sequences per sample was performed to normalize data for subsequent analysis.

### Dogs

There were no differences in community membership (Jaccard index, *P* = 0.084) or population structure (Yue & Clayton *P* = 0.41, Bray-Curtis *P* = 0.77). There were also no differences in richness, diversity and evenness between the different timepoints (all *P* > 0.05).

**Figure 1:**
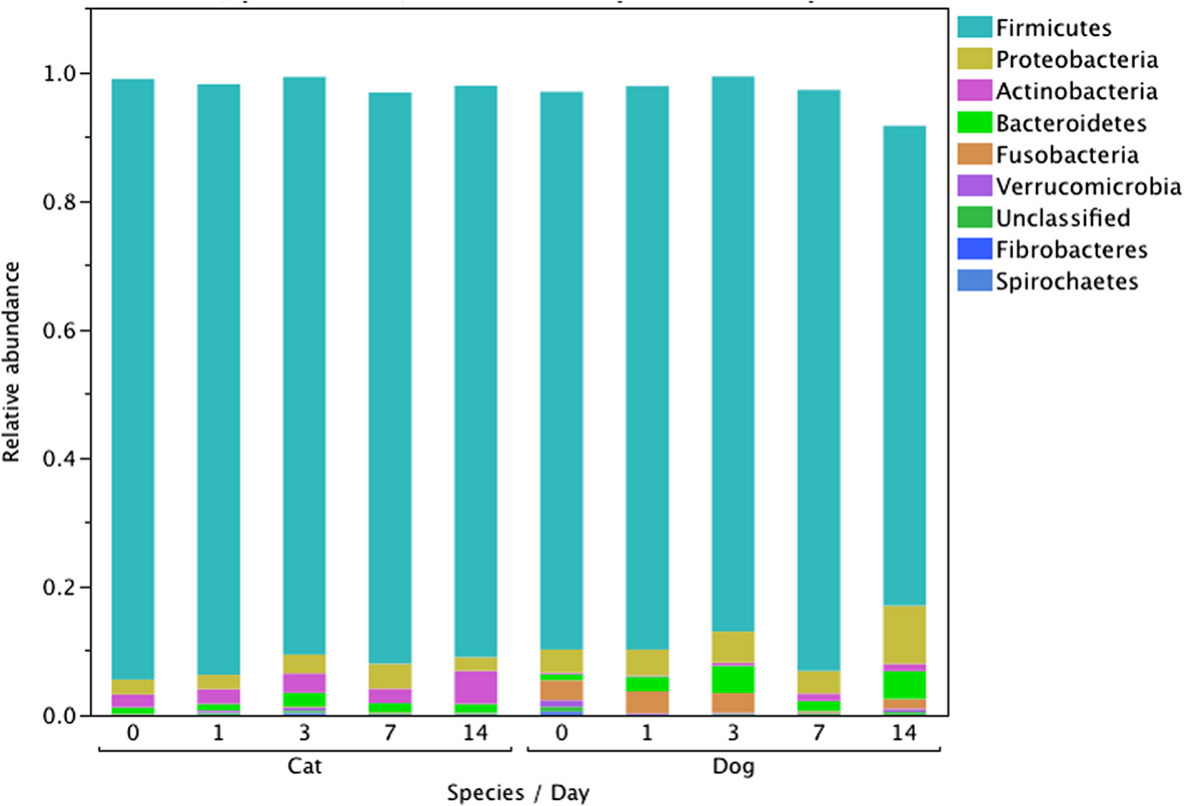
**Comparison of median relative abundances of predominant phyla of the fecal microbiota of dogs in samples tested after 0, 3, 7, 10 and 14 days of refrigeration.**

**Figure 2:**
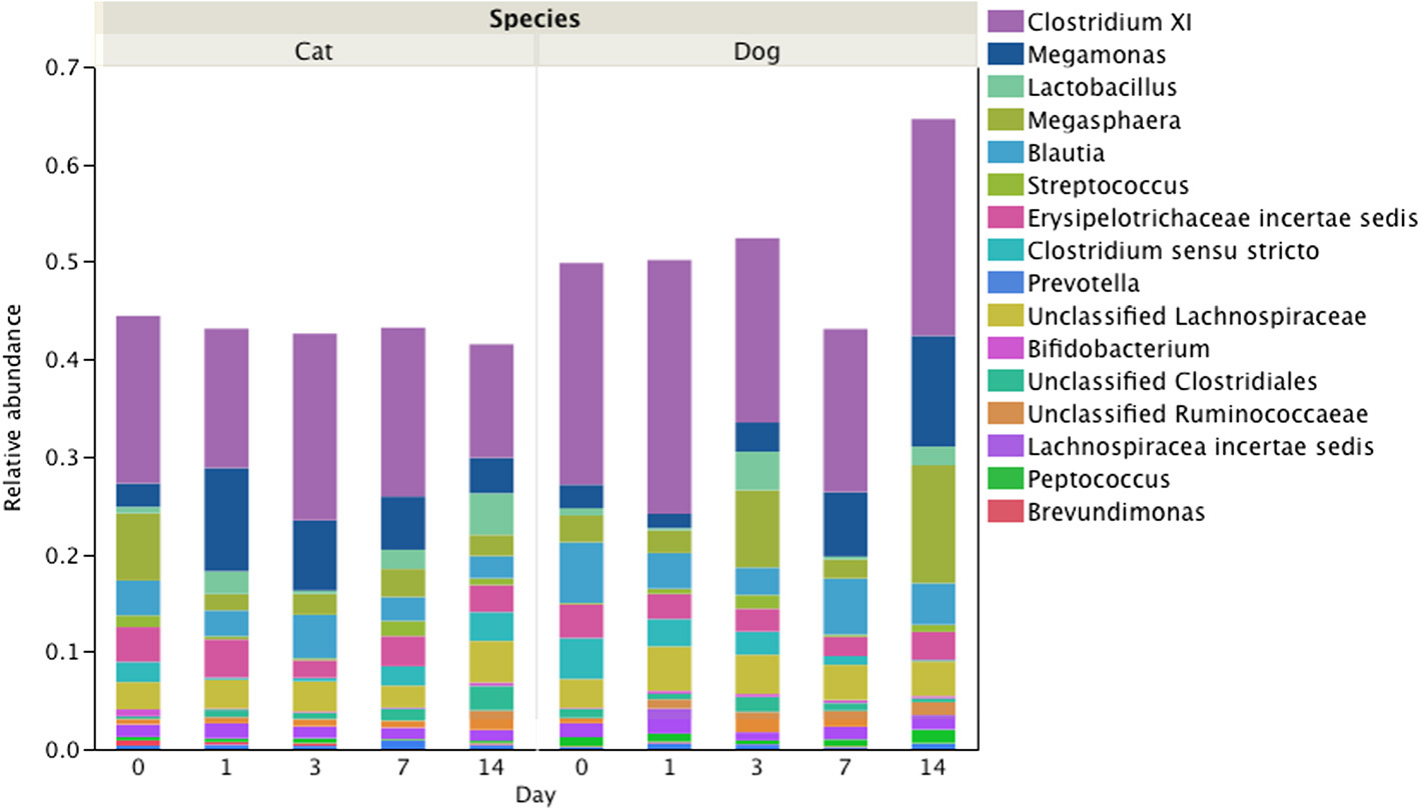
**Comparison of median relative abundances of genera accounting for at least 1% of the fecal microbiota of dogs in samples tested after 0, 3, 7, 10 and 14 days of refrigeration.**

Median relative abundances of phyla and genera accounting for 1% of more of the Day 0 or Day 14 microbiota are presented in Figures 1 and 2. Significant increases in Actinobacteria between Days 0-14 (median 0.23 vs 1.14%, *P =* 0.0184) and 1-14 (0.16 vs 1.14%, *P* = 0.0182) were identified. No significant differences were identified in relative abundance of predominant genera.

**Figure 3:**
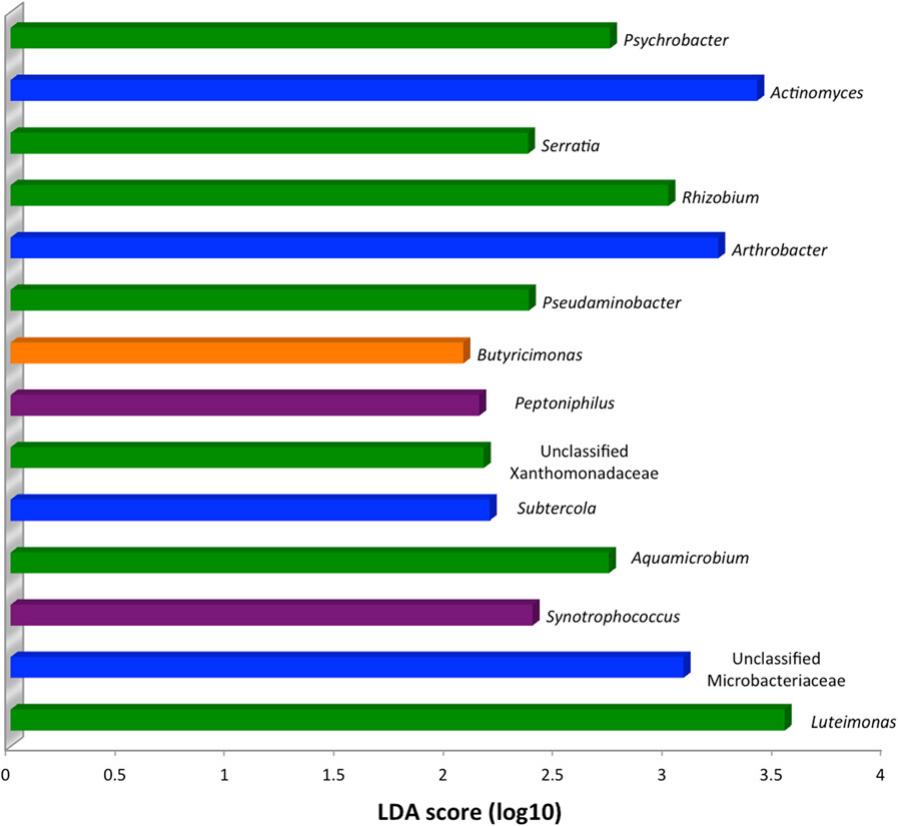
**LefSe results from the canine fecal microbiota indicating genera significantly associated with Day 14 (vs Day 0) samples.** Genera are colour coded by phylum. Orange: Bacteroidetes, Purple: Firmicutes, Green: Proteobacteria, Blue: Actinobacteria.

LEfSe identified 14 genera that were enriched in the Day 14 group compared to Day 0 (Figure 3). Four indicator OTUs were identified for Day 14 vs Day 0 samples, *Rhizobium, Psychrobacter, Serratia* and *Delftia*, all of which are Proteobacteria. Random forest modeling did not provide any further indication of an impact of storage time on the microbiota (data not presented).

### Cats

No differences in community membership (*P* = 0.24) or population structure (Yue & Clayton *P* = 1.0, Bray-Curtis *P* = 0.74) were identified by unifrac. There were also no differences in richness, evenness or diversity between groups.

No significant differences in phyla and predominant genera were identified, with the exception of a decrease in Erysipelotrichaceae *incerate sedis* between Day 0 and Day 7 (4.9 vs 2.2%, *P =* 0.046).

**Figure 4:**
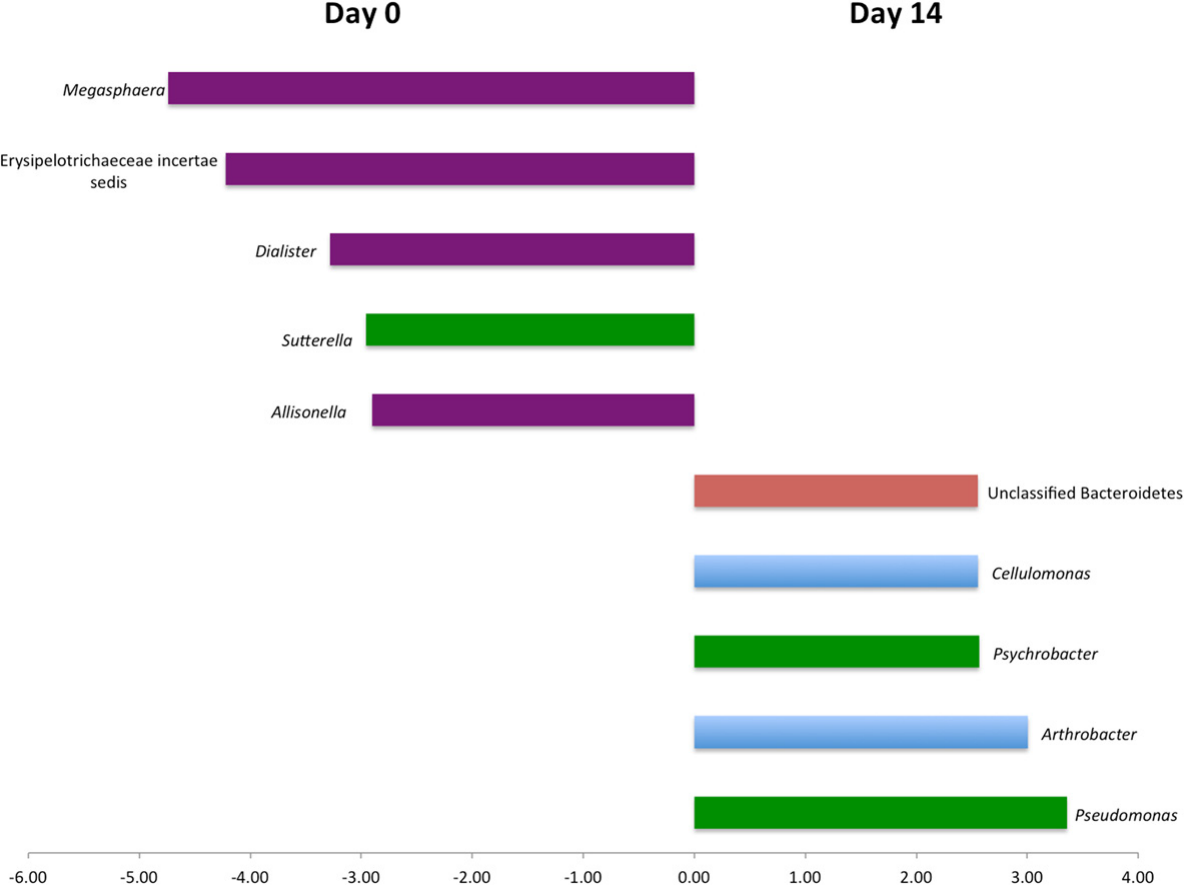
**LefSe results from the feline fecal microbiota indicating genera significantly associated with Day 14 and Day 0 samples.** Genera are colour coded by phylum. Orange: Bacteroidetes, Purple: Firmicutes, Green: Proteobacteria, Blue: Actinobacteria.

**Figure 5:**
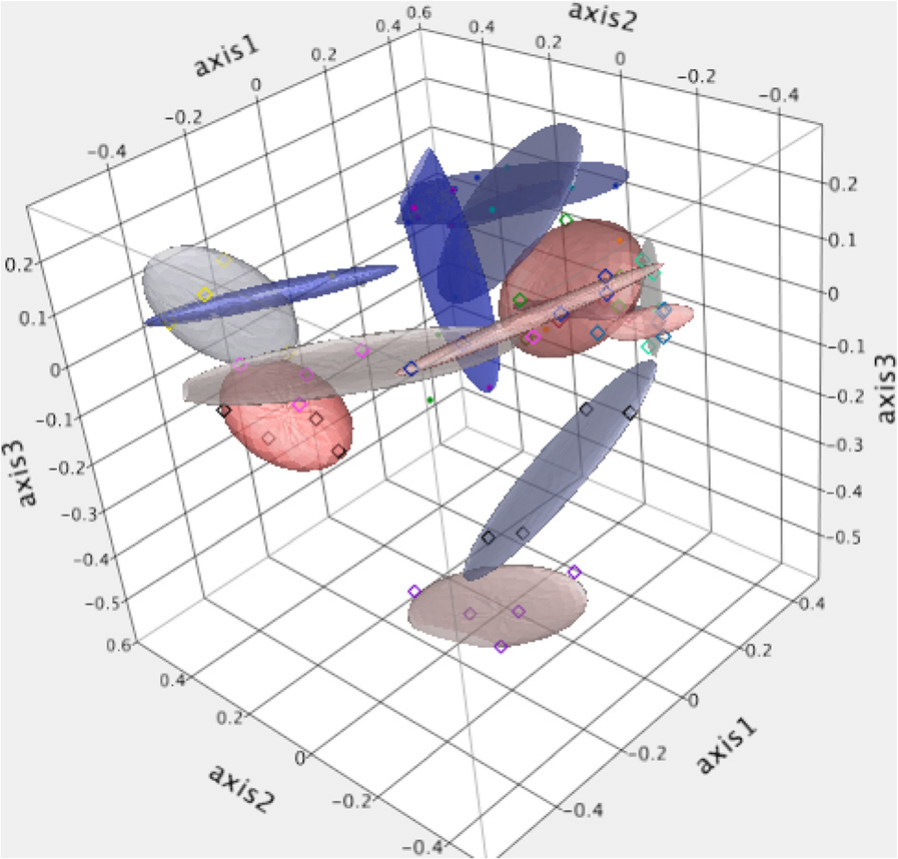
**Three dimensional principal coordinate analysis of the population structure of the fecal microbiota of cats (n = 12) after 0, 1, 3, 7 and 14 days of refrigeration.** Samples from individual cats are indicated by the same color and grouped by ellipses.

LEfSe results comparing Days 0 and 14 are presented in Figure 4. Indicator analysis showed a similar dominance of Proteobacteria and Actinobacteria as Day 14 indicators (Table 1). When Days 0 and 7 were compared, no significant indicators were identified and there were only two significant OTUs identified by LEfSe. These were both Firmicutes, with *Eubacterium* significantly associated with Day 0 and *Oscillospira* associated with Day 7. Principal coordinate analysis data are presented in Figure 5.

**Table 1:** **Indicator operational taxon units for the microbiota of feline fecal samples stored at 4°C**

**Day 0**	**Day 14**
*Allisonella* (Firmicutes)	*Brevundimonas* (Proteobacteria)
*Megasphaera* (Firmicutes)	*Arthrobacter* (Actinobacteria)
*Sutterella* (Proteobacteria)	*Slackia* (Actinobacteria)
	*Cellulomonas* (Actinobacteria)
	*Aeromicrobium* (Actinobacteria)
	*Phenylobacterium* (Proteobacteria)
	*Stenotrophomonas* (Proteobacteria)

## Discussion

Results of this study indicate that there are limited changes in the fecal microbiota of dogs and cats with short-term refrigeration. Most changes that were evident were identified at Day 14, with very little apparent impact of storage of seven days duration. Even by Day 14, changes were limited, with no differences in diversity, evenness and richness.

Various other microbiota assessment tools are available and consideration of the potential for differential impacts of storage is important. Assessment of the microbiota often involves evaluation ecological indices that evaluate OTUs that are present (membership) and OTUs that are present along with their relative abundances (structure). No alterations of these indices were noted. LEfSe is another method that is useful for analysis of high dimension data such as were present here, and which identifies OTUs that are most likely to explain differences between groups [16]. Canine samples after 14 days of storage were enriched in 14 genera, predominantly members of Actinobacteria and Proteobacteria. A similar pattern was noted in feline samples, with enrichment of certain Actinobacteria and Proteobacteria by Day 14. Two genera, *Psychrobacter* and *Arthrobacter,* were enriched in both canine and feline Day 14 samples.

Indicator analysis is an ecological tool that identifies members (in this case, OTUs) that ‘define’ a population, based on their presence and relative abundance. Indicator analysis yielded similar results to LEfSe, with three of the four genera identified as Day 14 indicators in dogs also identified by LEfSe. There was less agreement in of indicator analysis and LEfSe for feline samples, yet significant Day 14 results from both methods were dominated by Proteobacteria and Actinobacteria.

The increase in certain Proteobacteria and Actinobacteria was presumably a result of growth of these members during storage, as opposed to loss of other components. *Psychrobacter* can grow at a wide range of temperatures, including 4°C [19], something that likely accounts for its increased presence in both canine and feline Day 14 samples. A study of stored soil samples reported over-representation of Rhizobiales, Alphaproteobacteria and Thermomicrobia after refrigeration [9], some of which were also identified here.

Despite the changes that were noted, there was limited overall impact on the microbiota with fourteen days of refrigeration. However, these data suggest that consideration should be given to any lower level (e.g. genus) taxonomic differences that are noted in samples that have undergone storage, particularly those involving Proteobacteria and Actinobacteria.

This study evaluated dogs from an animal shelter. While historical data for these animals are limited, that should have limited impact on the objectives of this study, since it was designed to evaluate the effects of storage, not compare groups or define the canine microbiota. This study only evaluated samples for 14 days, so no assurance can be given that more profound changes do not occur with longer storage. Yet, it is reasonable to assume that samples could be processed or frozen within this timeframe, even with multicenter and field studies. The relatively small sample size that could have hampered identification of some differences. Another factor that should be considered is the potential for intra-sample variation. Another consideration is that if fecal samples are non-homogenous, some minor changes could potentially be the result of inherent intra-sample variation, an area that has not been carefully studied.

Nonetheless, there were few changes identified in the fecal microbiota in samples stored at 4C for 14 days, something that is consistent with previous studies of different sample types and storage conditions [6-9].

## Conclusion

Short term (up to 14 day) refrigeration should have limited impact on studies of the canine and feline fecal microbiota. However, the potential for changes in some members of the microbiota must be considered during study design and interpretation, particularly when there will be a focus on individual genera, as opposed to broader population-based analysis. Thus, it appears to be reasonable to use short term storage of canine and feline fecal samples, if required, but to strive for similar storage conditions between groups to remove any potential impact on subsequent analyses.

### Availability of supporting data

The dataset supporting the results of this article is available at the MG-RAST metagenomics analysis server (project 9714, http://metagenomics.anl.gov).

## Endnotes

^a^E.Z.N.A. Stool DNA Kit, Omega Bio-Tek Inc., Doraville, Georgia, USA.

^b^Nanodrop, Roche, Mississauga, Canada.

^c^Beckman Coulter Inc., Mississauga, Canada.

^d^Illumina, San Diego, USA.

## Abbreviations

LEfSe: Linear discriminant analysis effective size
OTU: Operational taxon unit
PCoA: Principle coordinate analysis

## References

[1] Garcia-Mazcorro JF, Dowd SE, Poulsen J, Steiner JM, Suchodolski JS (2012). Abundance and short-term temporal variability of fecal microbiota in healthy dogs. Microbiol Open.

[2] Handl S, German A, Holden S, Dowd S, Steiner J, Heilmann R, Grant R, Swanson K, Suchodolski J (2013). Faecal microbiota in lean and obese dogs. FEMS Microbiol Ecol.

[3] Suchodolski JS, Dowd SE, Wilke V, Steiner JM, Jergens AE (2012). 16S rRNA gene pyrosequencing reveals bacterial dysbiosis in the duodenum of dogs with idiopathic inflammatory bowel disease. PLoS One.

[4] Sturgeon A, Pinder SL, Costa MC, Weese JS: **Characterization of the oral microbiota of healthy cats using next-generation sequencing.** *Vet J*. doi:10.1016/j.tvjl.2014.01.024. 10.1016/j.tvjl.2014.01.024 24680670

[5] Rodrigues Hoffmann A, Patterson AP, Diesel A, Lawhon SD, Ly HJ, Elkins Stephenson C, Mansell J, Steiner JM, Dowd SE, Olivry T, Suchodolski JS (2014). The skin microbiome in healthy and allergic dogs. PLoS One.

[6] Carroll IM, Ringel-Kulka T, Siddle JP, Klaenhammer TR, Ringel Y (2012). Characterization of the fecal microbiota using high-throughput sequencing reveals a stable microbial community during storage. PloS One.

[7] Bai G, Gajer P, Nandy M, Ma B, Yang H, Sakamoto J, Blanchard MH, Ravel J, Brotman RM (2012). Comparison of storage conditions for human vaginal microbiome studies. PloS One.

[8] Lauber CL, Zhou N, Gordon JI, Knight R, Fierer N (2010). Effect of storage conditions on the assessment of bacterial community structure in soil and human-associated samples. FEMS Microbiol Lett.

[9] Rubin BER, Gibbons SM, Kennedy S, Hampton-Marcell J, Owens S, Gilbert JA (2013). Investigating the impact of storage conditions on microbial community composition in soil samples. PLoS One.

[10] Caporaso JG, Kuczynski J, Stombaugh J, Bittinger K, Bushman FD, Costello EK, Fierer N, Peña AG, Goodrich JK, Gordon JI, Huttley GA, Kelley ST, Knights D, Koenig JE, Ley RE, Lozupone CA, McDonald D, Muegge BD, Pirrung M, Reeder J, Sevinsky JR, Turnbaugh PJ, Walters WA, Widmann J, Yatsunenko T, Zaneveld J, Knight R (2010). QIIME allows analysis of high-throughput community sequencing data. Nat Methods.

[11] Schloss PD, Westcott SL, Ryabin T, Hall JR, Hartmann M, Hollister EB, Lesniewski RA, Oakley BB, Parks DH, Robinson CJ, Sahl JW, Stres B, Thallinger GG, Van Horn DJ, Weber CF (2009). Introducing mothur: open-source, platform-independent, community-supported software for describing and comparing microbial communities. Appl Environ Microbiol.

[12] Quast C, Pruesse E, Yilmaz P, Gerken J, Schweer T, Yarza P, Peplies J, Glöckner FO (2013). The SILVA ribosomal RNA gene database project: improved data processing and web-based tools. Nucleic Acids Res.

[13] Edgar RC, Haas BJ, Clemente JC, Quince C, Knight R (2011). UCHIME improves sensitivity and speed of chimera detection. Bioinformatics.

[14] **RDP.** [http://rdp.cme.msu.edu/index.jsp]

[15] Gihring TM, Green SJ, Schadt CW (2012). Massively parallel rRNA gene sequencing exacerbates the potential for biased community diversity comparisons due to variable library sizes. Env Microbiol.

[16] Segata N, Izard J, Waldron L, Gevers D, Miropolsky L, Garrett WS, Huttenhower C (2011). Metagenomic biomarker discovery and explanation. Genome Biol.

[17] Dufrêne M, Legendre P (1997). Species assemblages and indicator species: the need for a flexible asymmetrical approach. Ecol Monogr.

[18] Lozupone C, Hamady M, Knight R (2006). UniFrac–an online tool for comparing microbial community diversity in a phylogenetic context. BMC Bioinformatics.

[19] De Filippis F, La Storia A, Villani F, Ercolini D (2013). Exploring the sources of bacterial spoilers in beefsteaks by culture-independent high-throughput sequencing. PLoS One.

